# Coaching the Caregivers: A Burnout Intervention for Military Physicians and Medical Students †

**DOI:** 10.3390/ijerph23081087

**Published:** 2026-08-21

**Authors:** Lisa M. Foglia, Erin Keyser, Jason Massengill, Sorana Raiciulescu, Monica A. Lutgendorf

**Affiliations:** 1 Department of Obstetrics and Gynecology, Mountain Area Health Education Center, Asheville, NC 28803, USA; lisa.foglia@gmail.com; 2 Department of Graduate Medical Education, Brooke Army Medical Center, San Antonio, TX 78234, USA; doctorerin.cox@gmail.com; 3 Department of Graduate Medical Education, Boonshoft School of Medicine, Wright State University, Dayton, OH 45435, USA; jason.massengill@gmail.com; 4 Department of Preventive Medicine and Biostatistics, Uniformed Services University of the Health Sciences, Bethesda, MD 20814, USA; sorana.raiciulescu@usuhs.edu; 5 Department of Gynecologic Surgery and Obstetrics, Uniformed Services University of the Health Sciences, Bethesda, MD 20814, USA; 6 Department of Obstetrics and Gynecology, Division of Maternal-Fetal Medicine, Inova Health System, Fairfax, VA 22042, USA

**Keywords:** coaching, burnout, work–life integration

## Abstract

**Highlights:**

**Public health relevance—How does this work relate to a public health issue?**

- Physician burnout contributes to workforce attrition in obstetrics and gynecology, a specialty facing current and projected shortages that threaten access to maternity care and worsen maternity care deserts.
- Coaching may improve physician satisfaction, improve public health, enhance safety, and maintain a stable healthcare workforce.

**Public health significance—Why is this work of significance to public health?**

- Maintaining a healthy and engaged physician workforce is essential for ensuring access to high-quality maternal healthcare, particularly in the setting of OB/GYN physician shortages.
- This study identifies distinct burnout profiles among military OB/GYN physicians and demonstrates that professional coaching can improve perceptions of workload, control, and fairness among clinicians experiencing burnout.

**Public health implications—What are the key implications or messages for practitioners, policy makers and/or researchers in public health?**

- Healthcare organizations should consider professional coaching as part of a broader workforce well-being strategy to improve job satisfaction, organizational fit, and retention among physicians at risk for burnout.
- Policy makers and healthcare leaders should prioritize interventions that increase clinician autonomy, improve scheduling flexibility, reduce administrative burdens, and support meaningful work to sustain the maternal healthcare workforce and protect access to care.

**Abstract:**

Introduction: Burnout results from chronic stress, consisting of emotional exhaustion, depersonalization and decreased personal accomplishment. The objective of this study was to assess the effects of coaching on physician burnout in military obstetrician gynecologists (OB/GYNs). Methods: This was an IRB-approved observational study of coaching for military OB/GYN physicians and students. Physicians and medical students were assigned to attend six virtual group coaching sessions over three months with professional coaches. Coaching groups were organized by level of training. Participants completed surveys (Maslach Burnout Survey for Human Services Survey (MBI-HSS) with Areas of Worklife Survey (AWS)) at baseline and following coaching. A latent profile analysis (LPA) used MBI scores to identify burnout profiles in dimensions of depersonalization (DP), emotional exhaustion (EE), and personal accomplishment (PA). Statistical analysis included paired *t*-test, and chi squared analyses. Results: 59 participants completed coaching and surveys from August–October 2024. There were no significant differences in MBI scores pre- and post-coaching. LPA identified three profiles: (a) low DP, low EE, high PA, (b) low DP, moderate EE, high PA, (c) high DP, high EE, high PA. When analyzed on MBI definitions, those with a burnout profile (high DP and high EE) had significant improvements in workload, control, and fairness scores on the AWS following coaching (*p* < 0.05). Conclusions: A professional coaching intervention in military OB/GYN physicians with a burnout profile was associated with significant improvements in workload, control and fairness. Professional coaching may positively impact clinicians, particularly those experiencing burnout, and has the potential to improve retention of the workforce.

## 1. Introduction

The United States face a significant shortage of physicians, with the American Association of Medical Colleges (AAMC) projecting a shortage of 86,000 physicians by 2036 [1]. A 2021 report from the U.S. Department of Health and Human Services, Health Resources and Services National Center for Health Workforce Analysis states that practicing OB/GYNs in the US will decrease by 7% (from 50,850 to 47,490) while demand will increase of 4% (from 50,850 to 52,660) by 2030 [2]. This means that physician demand will exceed supply by 5170 FTEs in 2030 in the United States, with deficits projected in the West, South, and Midwest [2].

Such physician shortages are likely to further negatively affect maternity care deserts across the United States. Maternity care deserts remain an ongoing challenge, with one in 25 obstetric units closing from 2022 to 2024. Currently, over 35% of counties in the US are defined as a maternity care desert without a single birthing facility or obstetric clinician [3]. In these 1104 counties, over 2.3 million women of reproductive age do not have access to birthing facilities [3], and must travel longer distances, further exacerbating adverse social determinants of health and negatively impacting families with geographic and financial barriers to accessing care [3]. These inequities in maternity care primarily affect individuals in rural locations, as over 2/3 of maternity care deserts are in rural locations, and inequities exist based on race and healthcare coverage [3]. Maternity care deserts have been linked with suboptimal outcomes, including higher rates of maternal and pregnancy-related mortality [4], and increased infant mortality and low birthweight infants [5].

In light of ongoing shortages, concerns about physician well-being are increasing, with burnout affecting approximately 50% of physicians in the US [6]. OB/GYNs face unique challenges contributing to stress and leaving the profession early, such as long and unpredictable work hours, high-stress clinical environments, litigation concerns, financial pressures, and administrative burdens [7]. Specific OB/GYN stressors include caring for critically ill patients, patients and families at risk of fetal abnormalities, and facing high litigation risks which can further increase compassion fatigue and burnout [8]. Trainees and women may also be uniquely at risk for burnout. Burnout was higher in physicians in residency and fellowship compared to medical students, early career physicians, and the U.S. population [9]. A national survey of female resident physicians showed that 77% expressed emotional exhaustion with a strong association between emotional exhaustion and intent to leave training [10].

The military trains approximately 50 OB/GYN physicians annually through graduate medical education platforms. Military OB/GYNs provide care for 45,000 births annually across the United States and overseas, serving at bases in remote locations and urban centers. The number of military OB/GYNs has steadily declined, resulting in staffing shortages at many military facilities. Additionally, military OB/GYNs often shoulder increased leadership and teaching responsibilities alongside their clinical workload, while facing geographic separations. These factors may contribute to a heightened risk of burnout among military OB/GYNs. Furthermore, the effects of the COVID-19 pandemic may have heightened burnout in clinicians [11], particularly related to high levels of emotional exhaustion and depersonalization [11]. Although some studies note a decrease in burnout post-pandemic, levels remained higher than pre-pandemic levels and certain specialties such as primary care report the highest levels of burnout [12].

Interventions to improve burnout include primary prevention using structural workplace modifications such as reductions in workload [13] and secondary prevention such as mindfulness-based interventions [14], and enhancing individual coping and cognitive approaches through coaching. Individualized and group professional coaching may improve burnout in physicians, surgeons [15,16,17,18,19], and trainees [20,21,22]. However, a coaching intervention has not been studied in OB/GYNs, nor the military, which has unique stressors that may exacerbate burnout. The objective of this study was to determine the underlying level of burnout and assess the effectiveness of a group virtual coaching intervention on military physician burnout in obstetrics and gynecology. We hypothesized that a coaching intervention would improve participant scores on the MBI [23] and AWS [24] with improvements in the PCQ [25], PSS [26], FFWEL [27]. Table 1.

## 2. Materials and Methods

Military physicians, residents and students were recruited via email, social media blasts, and word of mouth to voluntarily join virtual group coaching sessions. Inclusion criteria included being a military medical student interested in OB/GYN (either attending the Uniformed Services University of the Health Sciences or participating in the Health Professions Scholarship Program at a civilian medical school), military OB/GYN residents in an OB/GYN residency, or an OB/GYN physician at a Military Treatment Facility, and interested in voluntarily participating in virtual coaching sessions. Exclusion criteria included civilian physicians employed by the MHS and those who declined to participate. Interested individuals were sent a study information sheet describing study participation and confidentiality, and if they agreed to participate, they emailed the principal investigator to be scheduled with a coaching group based on their level of training.

Following agreement to participate, they were assigned to one of four coaching groups based on their level of experience: medical student, resident physician, junior physician (<10 years from residency), and senior physician ≥ 10 years from residency. Two timeframes were offered for each level of coaching to maximize participation and accommodate participants across multiple time zones; thus, there was a total of 8 groups. Coaching groups included between 5 and 13 participants. Groups were self-selected based on the best time for participation based on their work and clinical schedules. The maximum number of participants in each group was limited to no more than 13 to allow for participation of all members. Virtual coaching sessions were conducted by four experienced professional physician coaches. Three of the four coaches also had experience with military medicine (prior service as military physicians). Coaching fees for the professional coaches were negotiated, and the cost was $750 per one hour group session, with a total of six sessions for 8 groups (48 sessions); this was $36,000. Coaching sessions were held on secured video conferencing. Sessions were scheduled for one hour, and participants were invited to a series of six coaching sessions over 12 weeks with the same group. Coaches determined the content of the coaching sessions tailored to their specific audiences and group interests. Coaching topics included resilience, boundary setting, applying for residency, managing workload and cognitive re-framing. A general list of topics covered during coaching sessions is described in more detail in Supplement S1.

Prior to the coaching intervention, participants completed an anonymous open-ended questionnaire that asked for input regarding what could improve wellness within their organization, what small changes would improve day to day work, barriers to efficiency, and observations about how people were treated that they perceived as inappropriate. Participants also completed five validated surveys prior to coaching, shown in Table 1. Participants were also asked demographic questions and open-ended questions related to their future career plans and programmatic feedback on the coaching experience (Supplement S2). Surveys were hosted on a secure online site by Mind Garden, Menlo Park, CA [23,24,25,26,27]. Participants received emails with login information, and all survey results were de-identified. Following the coaching sessions, participants completed the same surveys. Only participants who completed both pre- and post-coaching surveys were included in the data analysis.

The sample size was calculated using the null hypothesis, that the participants who undergo virtual coaching will have no difference in MBI scores, and the alternate hypothesis that participants who undergo coaching will have a difference in MBI scores. We hypothesized that MBI scores would be lower in participants following the coaching intervention due to improved skills at handling challenges and improved resilience. The prevalence of burnout in national physician samples is 50–60% [6,28], and to demonstrate a decrease of 58% in burnout from 60% to 35%, with 80% power and a two-tailed α = 0.05, we calculated we would need 62 participants. To account for dropouts, we planned to enroll 80 participants.

The primary outcome was the prevalence of burnout as measured by the MBI-HSS(MP). Secondary outcomes included changes in AWS, PCQ, PSS, and FFWEL survey scores. MBI-HSS(MP), AWS, PCQ, PSS and FFWEL survey results were compared pre- and post-coaching using a paired *t*-test. Significant results were then explored for participants who completed less than or equal to three sessions or greater than three sessions of coaching using repeated measures ANOVA. Subsequent between- and within-group pairwise comparisons were adjusted for multiple comparisons using the Sidak method. Tests were completed as two-sided tests, with *p* values of <0.05 considered statistically significant. Statistical analysis was completed using IBM SPSS Statistics version 30 for Windows, Chicago, Illinois.

Results from the pre-intervention MBI-HSS(MP) survey were used to conduct latent profile analysis [29] to identify groups of participants with similar patterns of burnout variables and to identify person-centered profiles across the burnout-engagement continuum. Mean values of emotional exhaustion (EE), depersonalization (DP) and personal accomplishment (PA) were used in the creation of up to five profile solutions. Model fit at each iteration was assessed using the Akaike Information Criterion (AIC), corrected Akaike Information Criterion (AICc), Bayesian Information Criterion (BIC), and Vuong–Lo–Mendell–Rubin test (VLMR) [30,31,32] for assessing improvement with an additional profile compared to the step beforehand, values of entropy that indicate greater, and class probabilities. A final solution of three profiles was chosen because of favorable and interpretable class probabilities. This analysis was completed using Stata Statistical Software: Release 19. College Station, TX, USA: StataCorp LLC.

Qualitative responses to the open-ended questionnaire were analyzed to assess common themes related to desired improvements and wellness. Themes were coded by LMF, with review and agreement among all authors. Analysis was conducted using thematic analysis [33]; themes were coded by hand and organized using a Microsoft Excel spreadsheet. This study was approved by the institutional review board as an exempt protocol (DBS.2024.665, 14 February 2024) and conforms with the U.S. Federal Policy for the Protection of Human Subjects.

## 3. Results

**Figure 1:**
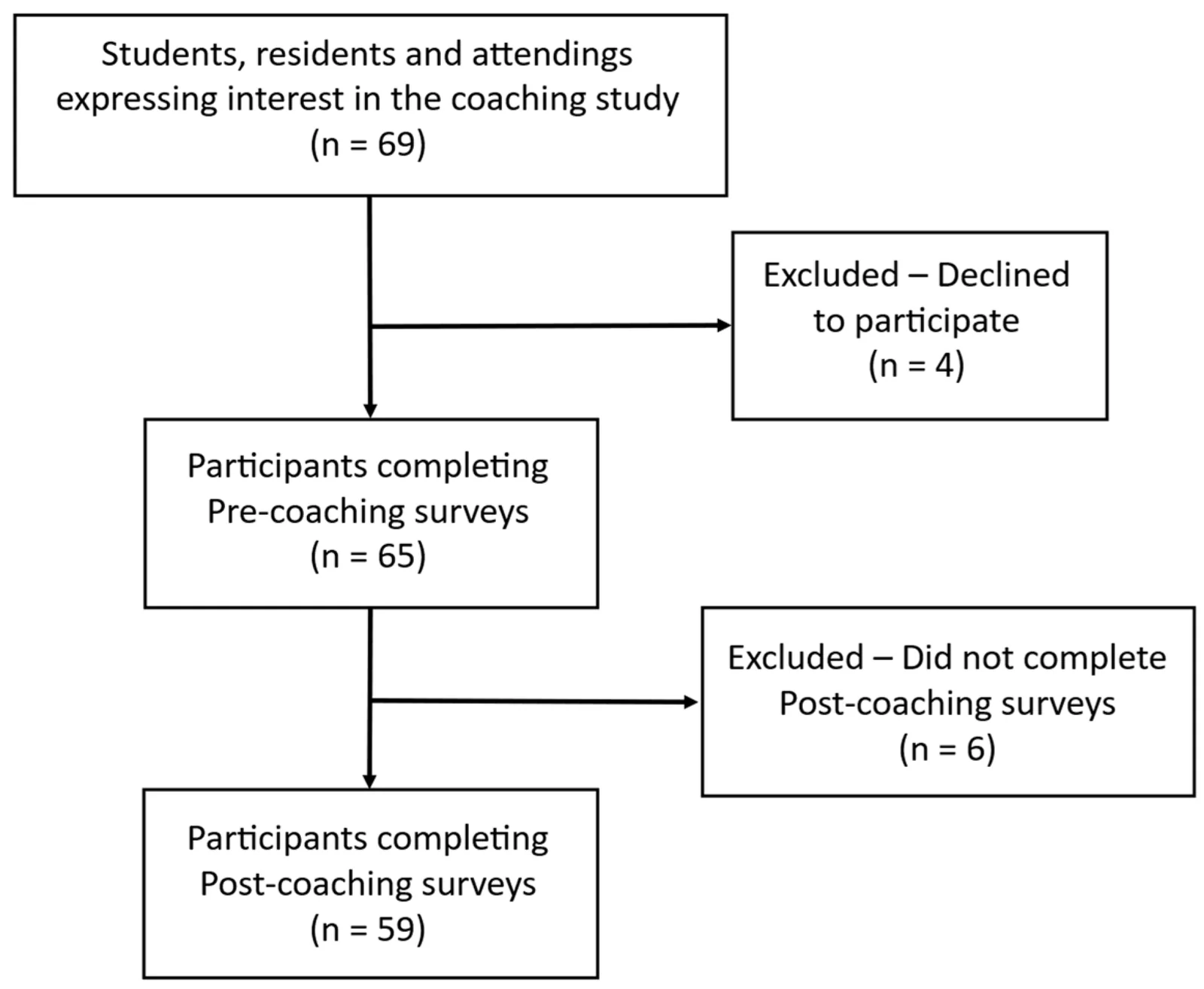
Flowchart of participants in the coaching study.

A total of 65 participants enrolled from 8 April 2024 to 20 June 2024, with 59 participants completing both surveys (Figure 1). Most participants were junior or senior faculty physicians, with residents comprising the smallest number of participants. On average, participants attended three to four of the six coaching sessions mean (4.17) SD (1.75) Most participants identified as White, and time in the military and time in role correlated with level of training (Table 2). Six participants did not complete post-coaching surveys, and were not included in the analysis. A sensitivity analysis was conducted comparing the baseline demographics of participants who dropped out of the study to those who completed the study, and there were no significant differences at the 0.05 level.

There were no significant differences in MBI-HSS(MP) average scores for emotional exhaustion, depersonalization, and personal accomplishment post-coaching compared to pre-coaching (Table 2). For AWS, there was a significant increase post-coaching scores in the domains of: workload, 2.61 pre-coaching vs. 2.85 post-coaching, *p* = 0.005, 95%CI 0.07–0.39; control, 3.2 pre-coaching vs. 3.5 post-coaching, *p* = 0.002, 95%CI 0.11–0.50; and fairness, 3.14 pre-coaching vs. 3.41 post-coaching, *p* = 0.001, 95%CI 0.11–0.43. For PCQ, there was a significant increase in efficacy scores post-coaching, 4.56 pre-coaching vs. 4.84 post-coaching, *p* = 0.002, 95%CI 0.10–0.45. There was no significant difference in PSS scores post-coaching compared to pre-coaching. On the FFWEL, there was a significant increase in physical self domain, 73.09 pre-coaching vs. 76.53 post-coaching, *p* = 0.02, 95%CI 0.46–6.40. (Table 3).

When the significant unadjusted findings were assessed using a model adjusting for number of coaching sessions attended, significant differences between pre- and post-coaching scores remained for participants completing three or more coaching sessions for AWS in the domain of workload, 2.57 pre-coaching vs. 2.85 post-coaching, *p* = 0.002, 95% CI 0.11–0.46, control 3.16 pre-coaching vs. 3.47 post-coaching, *p* = 0.004, 95%CI 0.10–0.52, and fairness 3.09 pre-coaching vs. 3.38 post-coaching, *p* = 0.002, 95%CI 0.11–0.47. A significant difference was seen in the PCQ efficacy scores, 4.59 pre-coaching vs. 4.87 post-coaching, *p* = 0.005, 95%CI −0.30–0.72, Table 3. There were no significant differences in FFWEL regardless of the number of coaching sessions attended (Table 4).

**Figure 2:**
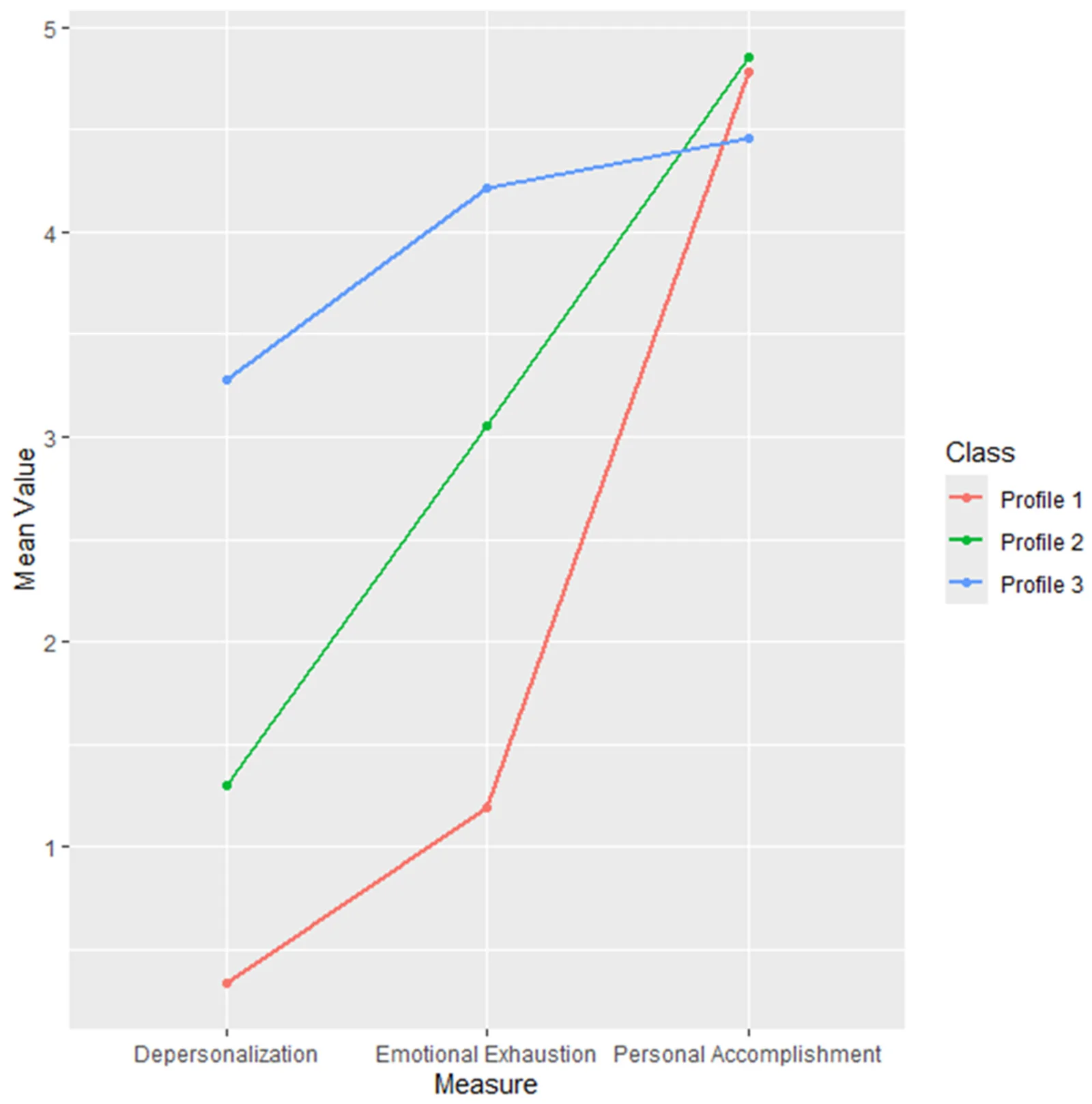
Plot of means for the 3-profile latent profile analysis. Profile 1 = engaged, Profile 2 = overextended, Profile 3—burnout.

Latent profile analysis (LPA) was used to identify groups of participants with similar patterns of burnout variables on initial surveys. Using LPA allows for the assessment of unique patterns in participants, allowing for an individualized assessment of the three dimensions for different people and identifying unique subpopulations [29]. Mean scores on DP, EE, and PA were used to construct a series of nested LPA models to determine the optimal number of clusters, starting with a single class model and progressing with the addition of an additional class until there was no improvement in the model fit [29]. When evaluating the model selection metrics, all solutions had high entropy (range 0.82–0.88), indicating the models had appropriate ability to differentiate participants into different profiles. As these are probabilistic models, individuals have predicted probabilities of placing into the respective number of profiles. More favorable models will show a high probability of membership into just one of the profiles. All solutions had similar values when assessed by Akaike Information Criterion (AIC), corrected AIC (AICc)), and Bayesian Information Criterion (BIC) (Supplement S3). The Vuong–Lo–Mendell–Rubin (VLMR) test showed additional value only with the addition of the fifth profile. Collectively, the highest importance was placed on the latent class probabilities and utility of the resulting marginal means by profile. While the 5-profile solution has a significant VLMR, indicating improvement from the 4-profile solution, the minimum class probabilities were quite low, which would lead to difficult interpretation and generalization. Therefore, the 3-profile model was preferred to prevent clinical over-stratification. For these reasons, the model with three profiles was chosen, with the first profile consistent with being engaged (low DP, low EE, and high PA), the second profile consistent with being overextended (low DP, mid EE, and high PA), and the third profile consistent with burnout (high DP, high EE, and high PA) (Figure 2). All three profiles demonstrated high PA, Figure 2.

Participant MBI-HSS(MP) mean scores were aggregated based on level of training into two groups, divided into trainees (students and residents) and faculty (junior and senior faculty) to assess differences between groups, and compared pre-coaching and post-coaching, as shown in Supplement S4. Trainees had higher rates of engagement than faculty at baseline, but faculty engagement increased after coaching; Supplement S4.

Several common themes emerged from the open-ended responses such as a desire for improved efficiency in the electronic health record, limitations in adequate number and/or training of support staff resulting in administrative/non-physician duties that need to be done by physicians due to lack of adequate number or adequately trained support staff. Staffing challenges were cited frequently, including hiring delays, staff turnover, and lack of physician control over their schedules. Regarding personal wellness, participants cited lack of protected time for adequate exercise, nutrition, and well-being, particularly in comparison to the rest of the military personnel who are provided time during the duty day to meet mandated physical fitness requirements and government service personnel who also may have protected time for exercise. Staffing issues were noted to impact patients by affecting access to care, timeliness for appointments/surgical procedures and inefficient communication systems.

## 4. Discussion

Following a virtual professional coaching intervention for military OB/GYN physicians and trainees, we did not see significant changes in MBI-HSS(MP) scores, though we noted significant small improvements in the AWS scores in domains of workload, control and fairness, indicating small improvements in the congruence of clinician perceptions of their job environments in these areas. Since coaches included boundary setting and cognitive re-framing in coaching groups, it is possible that these techniques provided new tools for participants to use in their professional lives, and that these approaches may have affected participant survey responses. Such potential improvements in cognitive framing and coping strategies may be important areas that individuals can use when faced with challenges. However, it is also critical that healthcare organizations recognize the importance of improving and correcting systemic issues to mitigate negative effects on clinicians and improve system functioning.

There was also a significant small increase in the PCQ for efficacy. This indicates a potential association of coaching with improvements in job congruence and psychological capital. Job congruence, or alignment of professional skills with the values and culture of their profession and organization creates improved satisfaction, well-being and performance [34]. In prior studies, using the AWS among social service workers, researchers found that using a setting-based approach to addressing workplace issues such as work environment, values, and control of work-related tasks and workload was more effective than focusing on lifestyle factors such as work–life balance and self-care [35]. Psychological capital is defined as four psychological states that improve work performance: hope, efficacy, resilience, and optimism [36]. Cultivating these areas in the workplace facilitates improved organizational performance, and empowers employees to lead more productive lives and create environments where employees and organizations can thrive [36,37]. Psychological capital can be used to tap into human potential for improvement and well-being, particularly in high stress environments with uncertainty, such as healthcare [37].

This study highlights several key points that leadership should consider when addressing burnout. The coaching intervention demonstrated an improvement in participants’ sense of control, which aligns with findings from Shanafelt et al. [38], that emphasize control and flexibility as one of nine organizational strategies to promote engagement and reduce burnout. Importantly, providing control and flexibility does not need to conflict with broader organizational goals. Simple measures such as protected time, flexible hours, or control over call schedules can significantly enhance physician satisfaction and reduce burnout while meeting the mission. Feedback from participants in our study further supports these findings. Barriers to efficiency included “out-of-work interests” and “poor scheduling,” while recommended changes included providing more surgical opportunities, suggesting the importance of aligning work with individual interests. When asked what would promote wellness, participants highlighted “less micromanagement” and “control over schedule,” reinforcing the need for autonomy and flexibility. Involvement of healthcare systems and leadership is a critical step toward addressing macro-level workplace issues such as administrative burdens, support staff shortages, and scheduling issues. While individual coaching can address personal responses to challenges, and empower physicians to speak up, durable changes need support and investments from healthcare systems.

When comparing trainees (students and residents) to practicing physicians (junior and senior faculty), we identified that although trainees had higher levels of engagement with the initial surveys, the faculty engagement increased following coaching sessions. While we cannot determine if this may be due to increased faculty participation in coaching or other confounding factors, this difference is interesting particularly considering the differences in duties and responsibilities in the healthcare system and suggests that interventions, including coaching, should be tailored to healthcare professionals’ needs.

We also found a similar pattern of burnout to a prior study of military physicians which provides additional support that burnout patterns may be different in military physicians compared to civilian physicians [35]. Olapeju et al. [39] found that burnout was reduced when military physicians were more satisfied with their career paths and promotion plan, suggesting that military physicians have other military factors that affect burnout. The qualitative responses found that clinicians struggled with inadequate support and clinical staff, decreased resources and lack of work–life balance [39]. These findings were also noted in an earlier study of military physician teaching faculty, which noted that drivers of dissatisfaction included insufficient administrative support and emphasis on bureaucratic tasks [40]. We found high levels of personal accomplishment on the LPA for all three profiles, including the profile with high levels of EE and DP (Figure 2). Though we cannot determine the exact cause, we postulate that this finding may be related to a unique sociocultural aspect of serving in military medicine, or perhaps selection bias with highly motivated individuals self-selecting to participate in a coaching program.

Research on academic medicine faculty has identified young age, female gender, and generalist roles as factors that increase burnout risk [38,39,40]. These characteristics are common among military OB/GYNs, many of whom remain on active duty to complete their four-year service obligation after residency. The OB/GYN specialty has become increasingly female-predominant, mirroring broader trends in the field. Interestingly, prior studies found that physicians who spent at least 20% of their time on the aspect of work most meaningful to them experienced burnout at half the rate of those who spent less than 20% of their time on meaningful activities [38]. This approach aligns with broader priorities and suggests that identifying and supporting individual physicians’ passions (such as teaching research and patient care) can reduce burnout, improve satisfaction, enhance patient care metrics, and support mission success.

The military (and civilian healthcare systems) includes a hierarchical structure that can suppress individual control and autonomy. Such hierarchical structures can make it more challenging for individual participants to speak up and share opportunities for improvements within healthcare systems. Thus, it is important for healthcare systems to provide formal and informal avenues for clinicians to share concerns and ideas for system improvements. A proposed direct implementation pathway for the military health system (or other healthcare system) interested in improving physician autonomy, reducing administrative burden and increasing clinical efficiency includes convening a group of interested parties, assessing current administrative burdens, eliminating or re-designing low-value tasks and inefficiencies, increasing physician decision-making authority and involvement, optimizing use of technology, and monitoring and refining the system based on physician feedback and efficiency metrics. Using formal quality improvement techniques, such as FOCUS-PDSA (plan-do-study-act) [41] and lean six sigma [42], can provide valuable structure when improving processes.

In the open-ended responses, participants frequently cited administrative burdens and electronic health record (EHR) inefficiencies as sources of inefficiency and frustration. Burdensome EHR systems are frequently cited contributors to burnout, including excessive data entry, high volumes of messaging, and suboptimal useability resulting in additional workload beyond clinical hours [43]. Focused opportunities for improvements in the quality of data and alerts, and improvements in efficiency can reduce the burden on clinicians and improve EHR efficiency. Health systems are often initiating digital health and artificial intelligence solutions to automate documentation; however, this requires comprehensive training for effective integration, and structured technological training and digital literacy are often lacking [44]. Thus, organizations must work to ensure that well-being programs and digital improvements are accompanied by appropriate technological training and monitoring.

This study has several limitations. First, the participants were military OB/GYN physicians and trainees, which may limit the generalizability of the findings to the civilian sector or fields outside of OB/GYN. Additionally, all participants began the study with very high levels of personal accomplishment (PA), which may have skewed the burnout results to appear less severe (with more individuals having overextended (high EE, low to moderate DP, and low to moderate PA) or disengaged profiles (low to moderate EE, high DP, and low to moderate PA), rather than burnout profiles (high EE, high DP and low PA) [29] than they might in a broader population, or may be a factor unique to military physicians based on other aspects of their career (such as military service or educational service) beyond their role of providing of direct patient care. For varying reasons (such as time constraints, work and other commitments), most participants attended only four of the six coaching sessions (67%), and it is possible that attending all six sessions could have yielded different outcomes. Due to attrition, with some participants choosing not to complete post-coaching surveys, we were underpowered, with only 59 participants compared to the calculated 62 participants. Additionally, the sample size calculation was based on the burnout rate for attending physicians using national physician samples, and students and trainees may have different rates of burnout that would affect the calculated sample size. As this study was an observational study (with a pre- and post-analysis of a single group of physicians and students), we cannot determine causality. Furthermore, we cannot determine whether coaching or other environmental changes (or other confounding external factors) in the Military Health System may have affected participant responses on surveys.

Another limitation was the use of different coaches, curriculum, and topics between groups (Supplement S1) with potential variability in coaching styles which may have influenced results and differences between groups. Coaches independently planned and administered the sessions based on their expertise and what was most relevant to each group. While this may have introduced variability across groups, this practice best served the different career stages. However, the variability of coaching topics and coaching techniques may have limited our assessment of coaching efficacy. The diversity of participants included in this study was another potential limitation related to intergroup variation and lack of intervention standardization. While including diverse groups allowed us to provide coaching to students and physicians at different stages in their careers, students and residents face different challenges and experiences than physicians, and some of these differences may have impacted participation, with resident physicians having the lowest participation of all groups. Additionally, the lack of long-term follow up was another limitation, as the surveys were conducted soon after coaching, and the coaching sessions were over a relatively short period. Thus, we cannot determine if coaching over a longer time would increase effectiveness, or if any potential cognitive improvements in workload perception or self-efficacy may persist for longer periods of time following coaching. Finally, the process of self-referral to coaching may have selected participants who are not representative of the population.

## 5. Conclusions

Given the growing shortage of OB/GYNs, coupled with rising rates of burnout, further exploration of coaching to improve retention and job satisfaction is warranted. Military OB/GYN physicians cited lack of support, lower compensation and inefficiencies as reasons why physicians leave military service. Additionally, exploration of different patterns of burnout, and unique contributing and protective factors in OB/GYN warrants deeper examination. The trends we noted following a coaching intervention require additional confirmation in future multi-center randomized controlled trials in different practice settings. We recommend prioritizing talent management, meaningful work allocation, and addressing barriers such as scheduling inefficiencies and micromanagement to improve retention and sustain the workforce. By addressing these challenges, we aim to ensure that OB/GYNs remain engaged and supported in caring for our patients.

## Figures and Tables

**Table 1:** Validated surveys completed by participants before and after coaching.

Survey	Areas Assessed, Number of Questions	Domains, Detail
Maslach Burnout Inventory—Human services Survey for Health Personnel (MBI-HSS) [21]	Emotional Exhaustion (EE), 9 questions Depersonalization (DP), 5 questions Personal accomplishment (PA), 8 questions	Emotional overextension, exhaustion Unfeeling and impersonal responses toward those in one’s care Competence and achievement in work.
Areas of Worklife Survey (AWS) [22]	Degree of congruence between person and job environment, 28 questions	Workload, control, reward, community, fairness, values
Psychological Capital Questionnaire (PSS) [23]	Assesses the individual’s positive psychological state, 24 questions	Self-efficacy for challenging tasks, optimism about success in the future, perseverance toward goals, resilience
Perceived Stress Scale (PSS) [24]	Assesses perception of stress, 10 questions	
Five Factor Wellness Inventory (FFWEL) [25]	Assesses wellness, 91 questions	Creative self, coping self, social self, essential self, physical self

**Table 2:** Participant characteristics overall and by level of experience.

Characteristic		Level of Experience			
	Overall (*n* = 59)	Student (*n* = 15)	Resident (*n* = 6)	Junior Faculty (*n* = 18)	Senior Faculty (*n* = 20)
Number of coaching sessions attended, mean (SD)	4.17 (1.75)	3.87 (1.89)	2.83 (1.72)	4.78 (1.48)	4.25 (1.74)
Coaching success, mean (SD) Likert scale (1–5)	3.66 (1.04)	3.73 (0.96)	3.5 (1.05)	3.72 (1.23)	3.6 (0.99)
Race *, *n* (%)					
White	44 (74.6)	9 (60)	4 (66.7)	17 (94.4)	14 (70)
Black	4 (6.8)	2 (13.3)	1 (16.7)	-	1 (5)
Asian	3 (5.1)	2 (13.3)	-	1 (5.6)	-
Hispanic	4 (6.8)	1 (6.7)	-	-	3 (15)
Multiracial or other race	4 (6.8)	1 (6.7)	1 (16.7)	-	2 (10)
Years left in military, median (IQR)	4 (5)	7 (5)	5.5 (4)	4.5 (4)	2 (4)
Intent on remaining in the military beyond obligation? Yes, *n* (%)	32 (54.2)	11 (73.3)	4 (66.7)	9 (50)	8 (40)
Intent on remaining in the military through retirement? Yes, *n* (%)	36 (61)	8 (53.3)	2 (33.3)	8 (44.4)	18 (90)
Intent to complete fellowship training? *n* (%)					
Yes	23 (39)	10 (66.7)	5 (83.3)	8 (44.4)	-
No	21 (35.6)	5 (33.3)	1 (16.7)	7 (38.9)	8 (40)
Completed fellowship	15 (25.4)	-	-	3 (16.7)	12 (60)
How long have you worked in the MHS?, *n* (%)					
0–6 months	11 (18.6)	11 (7.3)	-	-	-
7–11 months	-	-	-	-	-
1–2 years	7 (11.9)	3 (20)	4 (66.7)	-	-
3–5 years	8 (13.6)	1 (6.7)	2 (33.3)	5 (27.8)	-
6–10 years	7 (11.9)	-	-	7 (38.9)	-
11–15 years	8 (13.6)	-	-	5 (27.8)	3 (15)
16–20 years	14 (23.7)	-	-	-	14 (70)
21+ years	4 (6.8)	-	-	1 (5.6)	3 (15)
How long have you been in your current role in the MHS? *n* (%)					
0–6 months	17 (28.8)	8 (53.3)	-	6 (33.3)	3 (15)
7–11 months	5 (8.5)	2 (13.3)	-	-	3 (15)
1–2 years	22 (37.3)	4 (26.7)	6 (100)	10 (55.6)	2 (10)
3–5 years	10 (16.9)	1 (6.7)	-	2 (11.1)	7 (35)
6–10 years	2 (3.4)	-	-	-	2 (10)
11–15 years	1 (1.7)	-	-	-	1 (5)
16–20 years	2 (3.4)	-	-	-	2 (10)
21+ years	-	-	-	-	-
Year in medical school, *n* (%)					
MS 1	-	1 (6.7)	-	-	-
MS 2	-	6 (40)	-	-	-
MS 3	-	4 (26.7)	-	-	-
MS 4	-	4 (26.7)	-	-	-
USUHS Medical School	-	7 (46.7)	-	-	-
HPSP Medical School	-	8 (26.7)	-	-	-
Year in residency, *n* (%)					
PGY-1	-	-	-	-	-
PGY-2	-	-	3 (50)	-	-
PGY-3	-	-	3 (50)	-	-
PGY-4	-	-	-	-	-
Years from residency graduation, median (IQR)	-	-	-	-	-
Practice type, *n* (%)					
Comprehensive OB/GYN	20 (52.6)	-	-	12 (66.7)	8 (40)
Subspecialty Fellow	3 (7.9)	-	-	3 (16.7)	-
Subspecialist	15 (39.5)	-	-	3 (16.7)	12 (60)
Subspecialist fellows (*n* = 4)					
Maternal-Fetal Medicine	1 (25)	-	-	1 (25)	-
Minimally Invasive Gynecology	1 (25)	-	-	1 (25)	-
Reproductive Endocrinology and Infertility	1 (25)	-	-	1 (25)	-
Urogynecology	1 (25)	-	-	1 (25)	-
Subspecialists (*n* = 15)					
Gynecologic Oncology	2 (14.3)			1 (33.3)	1 (9.1)
Maternal–Fetal Medicine	4 (28.6)			1 (33.3)	3 (27.3)
Minimally Invasive Gynecology	1 (7.1)			1 (33.3)	-
Reproductive Endocrinology and Infertility	3 (21.4)			-	3 (27.3)
Urogynecology	4 (28.6)			-	4 (36.4)

* Modified to White, Non-White for any analyses; SD—standard deviation; IQR = interquartile range.

**Table 3:** Survey results for participants pre-coaching and post-coaching.

Measure	Pre-Coaching Mean [SE]	Post-Coaching Mean [SE]	Difference (Post-Pre) Mean [SE]	*p*-Value
Maslach Burnout Inventory—Human Services (Medical Personnel) (MBI-HSS(MP))				
Emotional Exhaustion	3.15 [0.17]	3.03 [0.18]	−0.12 [0.13]	0.37
Depersonalization	1.84 [0.16]	1.75 [0.15]	−0.09 [0.14]	0.49
Personal Accomplishment	4.70 [0.11]	4.85 [0.10]	0.15 [0.10]	0.14
Areas of Worklife Survey (AWS)				
Workload	2.61 [0.09]	2.85 [0.09]	0.23 [0.08]	0.005
Control	3.20 [0.11]	3.50 [0.09]	0.30 [0.09]	0.002
Reward	3.44 [0.10]	3.60 [0.09]	0.16 [0.09]	0.08
Community	3.87 [0.08]	3.85 [0.08]	−0.02 [0.08]	0.76
Fairness	3.14 [0.10]	3.41 [0.08]	0.27 [0.08]	0.001
Values	3.52 [0.10]	3.63 [0.08]	0.11 [0.07]	0.11
Psychological Capital Questionnaire (PCQ)				
Hope	4.50 [0.09]	4.63 [0.10]	0.13 [0.08]	0.11
Efficacy	4.56 [0.11]	4.84 [0.10]	0.28 [0.09]	0.002
Resiliency	4.60 [0.08]	4.75 [0.08]	0.16 [0.08]	0.06
Optimism	4.12 [0.11]	4.22 [0.12]	0.09 [0.08]	0.22
Overall score	4.45 [0.55]	4.61 [0.56]	0.17 [0.06]	0.007
Perceived Stress Scale (PSS)	16.93 [0.82]	15.98 [0.80]	−0.95 [0.71]	0.19
Five Factor Wellness (FFWEL)				
Creative Self	78.45 [0.96]	79.28 [1.21]	0.82 [1.05]	0.44
Coping Self	70.89 [1.36]	71.43 [1.38]	0.54 [1.12]	0.63
Social Self	89.63 [1.37]	88.97 [1.53]	−0.69 [1.31]	0.60
Essential Self	75.20 [1.37]	76.83 [1.47]	1.64 [0.87]	0.07
Physical Self	73.09 [1.82]	76.53 [1.87]	3.43 [1.48]	0.02
Overall life satisfaction index	82.63 [2.03]	81.78 [2.17]	−0.85 [2.00]	0.67

SE = standard error of the mean.

**Table 4:** Survey results for participants’ pre-coaching and post-coaching, adjusted for number of coaching sessions attended.

	<3 Coaching Sessions				≥3 Coaching Sessions			
Measure	Pre-Coaching Mean [SE]	Post-Coaching Mean [SE]	Difference (Post-Pre) Mean [SE]	*p*-Value	Pre-Coaching Mean [SE]	Post-Coaching Mean [SE]	Difference (Post-Pre) Mean [SE]	*p*-Value
Areas of Worklife Survey (AWS)								
Worklife	2.82 [0.22]	2.82 [0.23]	0.00 [0.19]	1	2.57 [0.10]	2.85 [0.1]	0.28 [0.09]	0.002
Control	3.37 [0.27]	3.62 [0.21]	0.25 [0.23]	0.29	3.16 [0.12]	3.47 [0.10]	0.31 [0.105]	0.004
Fairness	3.39 [0.22]	3.54 [0.10]	0.15 [0.19]	0.44	3.09 [0.10]	3.38 [0.09]	0.29 [0.88]	0.002
Psychological Capital Questionnaire (PCQ)								
Efficacy	4.4 [0.26]	4.66 [0.23]	0.26 [0.21]	0.23	4.59 [0.12]	4.87 [0.11]	0.28 [0.10]	0.005
Five Factor Wellness (FFWEL)								
Physical Self	73.25 [4.45]	78.25 [4.57]	5 [3.63]	0.17	73.06 [2.01]	76.17 [2.06]	3.11 [1.64]	0.06

SE = standard error of the mean.

## Data Availability

The data that support the findings of this study are not publicly available due to ethical restrictions and participant privacy, and cannot be shared due to confidentiality agreements. Access is restricted to the authors and collaborators.

## Supplementary Materials

The following supporting information can be downloaded at https://www.mdpi.com/article/10.3390/ijerph23081087/s1, Supplement S1: Coaching topics discussed; Supplement S2: Additional questions; Supplement S3: Comparison of model fit for different profiles; Supplement S4: Maslach Burnout Inventory-Human Services (Medical Personnel) (MBI-HSS(MP)) for trainees and faculty pre-coaching and post-coaching.
