# Supplementary material for: Coaching the Caregivers: A Burnout Intervention for Military Physicians and Medical Students
Source: Int J Environ Res Public Health. 2026 Aug 21;23(8):1087. doi: 10.3390/ijerph23081087 (PMC13512648; doi:10.3390/ijerph23081087)
Supplement: Supplementary file 1 [file ijerph-23-01087-s001.zip › ijerph-4414774-supplementary.pdf]

Supplement S1—Coaching topics discussed

Coaching group	Topics Discussed
Medical Students	Residency application/match process<br>How to develop a competitive residency application<br>AAMC tools for self-awareness and specialty selection<br>Self-care<br>Priorities in school, life, etc.<br>Specialty disrespect<br>Research opportunities<br>How to address equity topics e.g., women in underrepresented fields<br>Rotations
Resident Physicians	Team cohesion, coping<br>Career options<br>Work–life balance
Junior Physicians (< 10 years from residency)	Cognitive Thought Model (CTFA). Circumstance thought leads to a feeling which leads to an action (propelling emotions vs. emotions that keep us stuck). Actions come from feelings and create results.<br>Give yourself permission to feel your feelings; then, you can decide if you want to continue to have those feelings.<br>Accept thoughts, don't judge. Your thoughts and feelings matter. Accept it before you work on fixing it. The smarter you are and the more operational you are, you are more likely to drift into black and white thinking. Thoughts are not the truth.<br>Shift your thought from negative to neutral.<br>Re-wire your brain to set boundaries.<br>Saying yes—think through—if I say yes, what am I saying no to! You don't want to be the person you disappointed at the end of your life.<br>Feelings behind procrastination.<br>Relaxation and meditation techniques.
Senior Physicians (≥ 10 years from residency)	Wheel of life assessment sheet<br>Complaining exercise<br>Induced thought model<br>Boundary setting: define, communicate, consequence<br>Facts vs. circumstances<br>Thought model (intentional and unintentional); things we can't control/change (e.g., big system undergoing lots of changes), if you can't change the system, you can adapt or leave. How do you convince people to do what you want? (negotiations)—Tactical empathy = trust and collaboration, influencing people by influencing their thoughts. Be clear about what you are thinking and feeling BEFORE you start talking. Use the thought model and use the part that includes you. Channel a thought—e.g., certainty (all problems fall into three buckets: 1. We know what it is and we can fix it, 2. We rule out bad things and feel safe, 3. We don't know what it is but we keep working to find a solution).

Supplement S2. Additional questions

Demographic and work questions included in the pre-survey

1. Please tell us your current role in the MHS:
2. What two or three things would promote wellness in this organization?
3. What are one or two small changes you'd like to make in your day-to-day work?

4. What keeps you from working efficiently?
5. What are the irritants in your job? “What is the pebble in your shoe?”
6. What do you see happening to patients/clients/customers which you don’t think should happen?
7. Please share your racial identity.
8. How long have you worked in the Military Health System?
 0-6 months
 7-11 months
 1-2 years
 3-5 years
 6-10 years
 11-15 years
 16-20 years
 21+ years
10. How long have you worked in your current position in the MHS?
 0-6 months
 7-11 months
 1-2 years
 3-5 years
 6-10 years
 11-15 years
 16-20 years
 21+ years
Role: student, resident, junior faculty (< 10 years from completion of residency), senior faculty (>/= 10 years from completion of residency)
15. Student, year in medical school ___
 USUHS
 HPSP
16. Resident, year in residency ___
17. Junior faculty (<10 years from completion of residency)
 Number of years from residency graduation ___
 Subspecialty fellow, year in fellowship ____
 Subspecialty fellowship in ____
 Comprehensive OB/GYN (generalist)
 Subspecialist, subspecialty _______
18. Senior faculty (>/= 10 years from completion of residency)
 Number of years from residency graduation ___
 Subspecialty fellow, year in fellowship ____
 Subspecialty fellowship in ____
 Comprehensive OB/GYN (generalist)
 Subspecialist, subspecialty _______
 Plans for practice in the MHS:
19. How many years do you have left on your military obligation? (or how many years will you have on your military obligation when you finish medical school, residency or fellowship?) ___
20. Do you intend to remain in the military beyond your obligation?
21. Do you currently intend to remain in the military through retirement?
22. Do you intend to complete fellowship training?

Programmatic questions included in the post-survey

1. How many coaching sessions did you attend?
2. Please rate the coaching program’s overall success at meeting your needs:

5—very helpful
4—Somewhat helpful
3—Unsure if it was helpful
2—Not really helpful
1—Very un-helpful

3. Please tell us what you liked best about the coaching program.
4. Please tell us what you liked least about the coaching program.
5. Please tell us what we should improve in the future.
6. Please provide any additional comments or feedback.

Supplement S3. Comparison of model fit for different profiles.

Solution	VLMR (p)	AIC	AICc	BIC	Latent class probabilities	Entropy
1 profile	-	554.92	556.54	567.39	-	-
2 profiles	0.06	524.15	528.73	544.93	0.39–0.61	0.84
3 profiles	0.72	520.73	530.28	549.82	0.18–0.47	0.82
4 profiles	0.20	518.34	535.44	555.74	0.16–0.35	0.85
5 profiles	0.01	499.91	528.02	545.61	0.02–0.26	0.88

Akaike Information Criterion (AIC), corrected AIC (AICc), and Bayesian Information Criterion (BIC), Vuong-Lo-Mendell-Rubin (VLMR) test.

Supplement S4. Maslach Burnout Inventory-Human Services (Medical Personnel) (MBI-HSS(MP)) for trainees and faculty pre-coaching and post-coaching

	Trainees (students and residents) (n = 21)		Faculty (junior and senior) (n = 38)	
Profile n (%)	Pre-Coaching	Post-Coaching	Pre-Coaching	Post-Coaching
Engaged	9 (42.9)	8 (38.1)	10 (26.3)	17 (44.7)
Ineffective	4 (19.0)	6 (28.6)	2 (5.3)	2 (5.3)
Overextended	3 (14.3)	3 (14.3)	14 (36.8)	11 (28.9)
Disengaged	1 (4.8)	1 (4.8)	1 (2.6)	0 (0)
Burnout	4 (19.0)	3 (14.3)	11 (28.9)	8 (21.0)

Engaged = Low emotional exhaustion, Low depersonalization. High personal accomplishment. Ineffective = Low-to-moderate emotional exhaustion. Low to moderate depersonalization. Low personal accomplishment. Overextended = High emotional exhaustion. Low to moderate depersonalization. Low-to-moderate personal accomplishment. Disengaged = Low-to-moderate emotional exhaustion. High depersonalization. Low-to-moderate personal accomplishment. Burnout = High emotional exhaustion. High depersonalization. Low personal accomplishment.
